# Second heart sound splitting as an indicator of interventricular mechanical dyssynchrony using a novel splitting detection algorithm

**DOI:** 10.14814/phy2.14687

**Published:** 2021-01-05

**Authors:** Hongxing Luo, Philip Westphal, Mehrdad Shahmohammadi, Luuk I. B. Heckman, Marion Kuiper, Richard N. Cornelussen, Tammo Delhaas, Frits W. Prinzen

**Affiliations:** ^1^ Department of Physiology Cardiovascular Research Institute Maastricht (CARIM Maastricht the Netherlands; ^2^ Bakken Research Centre Medtronic, plc Maastricht the Netherlands; ^3^ Department of Biomedical Engineering Cardiovascular Research Institute Maastricht (CARIM Maastricht the Netherlands

**Keywords:** cardiac dyssynchrony, heart sound, pacing therapy, S‐transform

## Abstract

Second heart sound (S2) splitting results from nonsimultaneous closures between aortic (A2) and pulmonic valves (P2) and may be used to detect timing differences (dyssynchrony) in relaxation between right (RV) and left ventricle (LV). However, overlap of A2 and P2 and the change in heart sound morphologies have complicated detection of the S2 splitting interval. This study introduces a novel S‐transform amplitude ridge tracking (START) algorithm for estimating S2 splitting interval and investigates the relationship between S2 splitting and interventricular relaxation dyssynchrony (IRD). First, the START algorithm was validated in a simulated model of heart sound. It showed small errors (<5 ms) in estimating splitting intervals from 10 to 70 ms, with A2/P2 amplitude ratios from 0.2 to 5, and signal‐to‐noise ratios from 10 to 30 dB. Subsequently, the START algorithm was evaluated in a porcine model employing a wide range of paced RV‐LV delays. IRD was quantified by the time difference between invasively measured LV and RV pressure downslopes. Between LV pre‐excitation to RV pre‐excitation, mean S2 splitting interval decreased from 47 ms to 23 ms (*p* < .001), accompanied by a decrease in mean IRD from 8 ms to −18 ms (*p* < .001). S2 splitting interval was significantly correlated with IRD in each experiment (*p* < .001). In conclusion, the START algorithm can accurately assess S2 splitting and may serve as a useful tool to assess interventricular dyssynchrony.

## Graphical abstract

We introduce an S‐transform amplitude ridge tracking (START) algorithm for estimation of 2nd heart sound (S2) splitting and investigated the relationship between S2 splitting and time difference in contraction between right and left ventricle in a porcine model. START was accurate in estimating S2 splitting interval in chirp‐model‐generated heart sounds. S2 splitting correlated well in pacing‐induced time differences in contraction of the ventricles in an animal model.

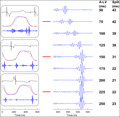

## 1 INTRODUCTION

Heart sounds, originating from vibrations of valves and adjacent tissues following valve closure, contain information of timing differences (dyssynchrony) of contraction and relaxation between the ventricles (Faber, 1964; Leatham, 1954; Luisada et al., 1973). Physiological splitting of the second heart sound (S2) occurs during inspiration when the difference between the timing of aortic and pulmonic valve closure is accentuated because the right ventricular (RV) ejection period is extended with a temporary increase in central venous return. Wide splitting is seen in conditions that delay RV emptying like right bundle branch block. Reverse splitting, that is, splitting during expiration, is associated, among others, with the left bundle branch block. Because of these relations between S2 splitting behaviors and ventricular activation patterns, quantification of S2 splitting might help to assess the degree of dyssynchrony in patients eligible for cardiac resynchronization therapy (CRT) and also to specify the settings of pacemakers that aim at minimizing dyssynchrony. However, currently heart sound indicator and algorithm to monitor interventricular dyssynchrony in these patients are lacking (Brugada et al., 2016; Taha et al., 2010; Toggweiler et al., 2006, 2007; Zuber et al., 2008).

Estimating heart sound splitting interval has long been a challenge because of the overlap of heart sound components. Auscultatory detection of S2 splitting is possible at intervals of at least 40 ms, whereas detection of intervals of 20–40 ms is only feasible with low ambient noise and extensive listener's experience (Al‐Naami et al., 2010). Importantly, this borderline area is where normal/physiological heart sound splitting occurs. Digital recording of heart sounds and subsequent analyses obviously avoid the limitations of the human ear and brain. Existing heart sound splitting detection algorithms can be mainly divided into three categories: 1) mathematical modeling of heart sound morphologies and inferring splitting interval by comparing morphology similarity between simulated and real heart sounds; 2) blind source separation using multiple‐channel simultaneous recordings; and 3) time–frequency analysis followed by visual identification of splitting heart sound components (Al‐Naami et al., 2010; Barma et al., 2015; Chen et al., 2018; Debbal & Bereksi‐Reguig, 2006, 2007; Djebbari & Bereksi‐Reguig, 2013; Nigam & Priemer, 2006; Popov et al., 2004; Thiyagaraja et al., 2014; Xu et al., 2002; Yildirim & Ansari, 2007). Modeling approaches have been dampened by the lack of a unified model of heart sound genesis (Popov et al., 2004; Xu et al., 2002). Blind source separation requires multi‐sensor recordings, hypothesis of heart sound transmission and complicated mathematical calculation (Chen et al., 2018; Nigam & Priemer, 2006). Time–frequency analysis approaches, such as continuous wavelet transform (CWT) and smoothed Wigner–Ville distribution (SWVD), have been popular in recent years (Al‐Naami et al., 2010; Barma et al., 2015; Debbal & Bereksi‐Reguig, 2006, 2007; Djebbari & Bereksi‐Reguig, 2013; Thiyagaraja et al., 2014; Yildirim & Ansari, 2007). However, CWT decomposes heart sounds into various scales, making further signal processing required to translate CWT results into a time–frequency spectrum. SWVD is undermined by its cross‐terms which may heavily interfere with the real heart sound components. As one of time–frequency analysis methods, S‐transform has been proposed as an energy‐concentrated signal processing approach which has a more direct relation with frequency compared with CWT and which has no cross‐terms interference compared with SWVD (Stockwell et al., 1996).

It is the aim of this study to develop a single‐heartbeat S2 splitting method that may be applied to CRT. Our S2 splitting measurement is based on S‐transform. We first validated the algorithm using a chirp model of simulated heart sound and subsequently applied the algorithm to an animal model of varying interventricular delays.

## 2 METHODS

### 2.1 S‐transform for signal analysis

**Figure 1:**
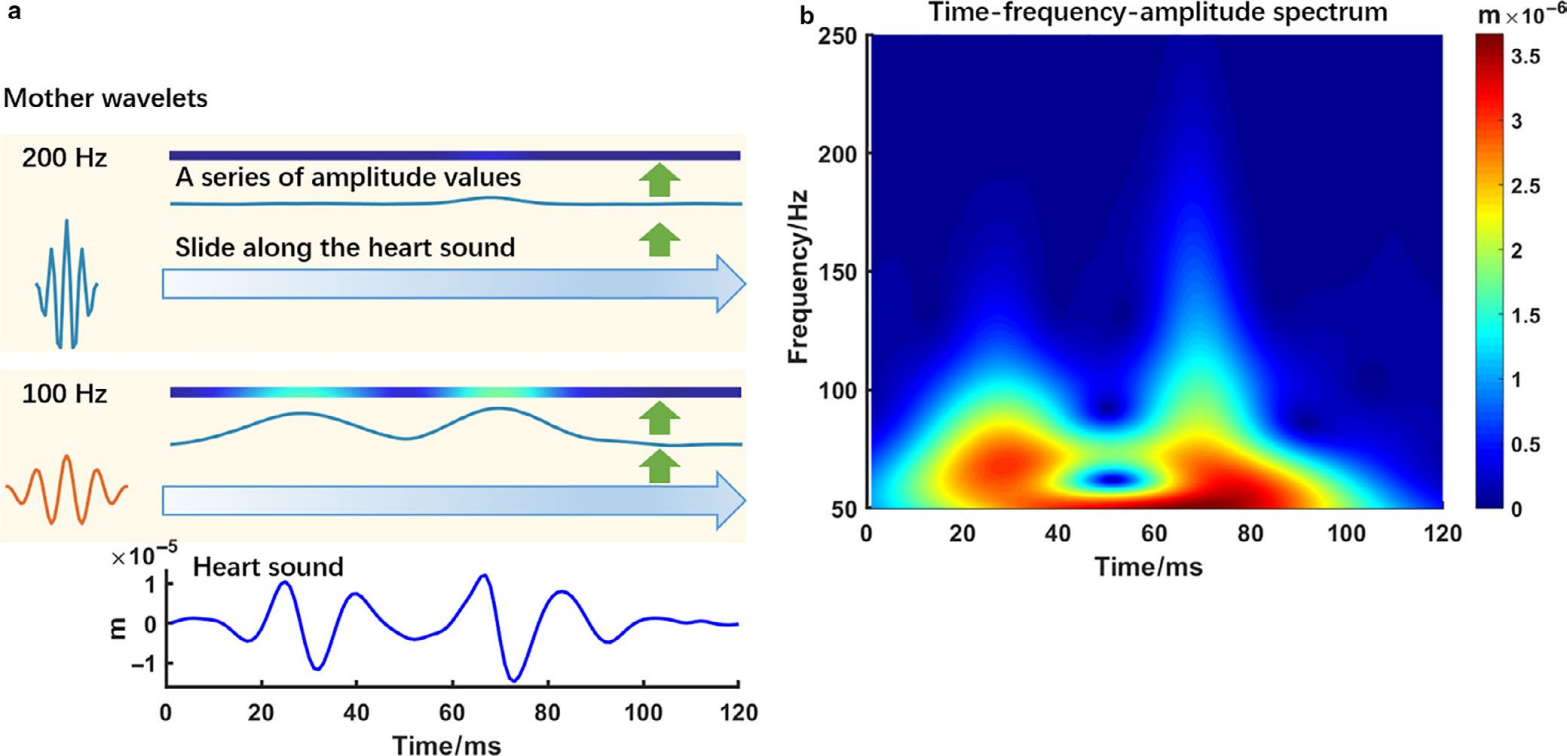
Demonstration of S‐transform for signal analysis using an S2 from an animal experiment. Panel A shows (from bottom to top) 1) the S2 during biventricular pacing with the right atrium to RV (A‐RV) paced interval 150 ms and A‐LV 50 ms, 2) a mother wavelet of 100 Hz and the resulting amplitude values with time, and 3) a mother wavelet of 200 Hz and the resulting amplitude values with time. Panel B shows the complete S‐transform amplitude spectrum. In panel A, the mother wavelets of various frequencies slide along the heart sound signal, convolve with the signal, and result in amplitude values. In panel B, amplitude values of frequencies from 50 Hz to 250 Hz are plotted as an intensity map

S‐transform is a signal processing technique used to analyze time–frequency features of a signal (Stockwell et al., 1996). It slides a mother wavelet of a given frequency along the raw signal (Figure 1a). Note that the width of the mother wavelet decreases with increasing frequency and height increases with frequency. For each time point, the wavelet is multiplied with the raw signal, and the resultant values are summed as an amplitude value. Similar to the principle of fast Fourier transform, this amplitude value would be high if the raw signal has a similar shape to the mother wavelet, and vice versa. This enables to extract the frequency contents of a signal using wavelets of a given range of frequencies, resulting in a matrix of amplitude values with time as the horizontal axis and frequency as vertical axis, or an S‐transform amplitude spectrum (Figure 1b). For our purpose, we used the frequency range of 50–250 Hz.

### 2.2 S‐transform amplitude ridge tracking (START)‐based detection of S2 splitting interval

**Figure 2:**
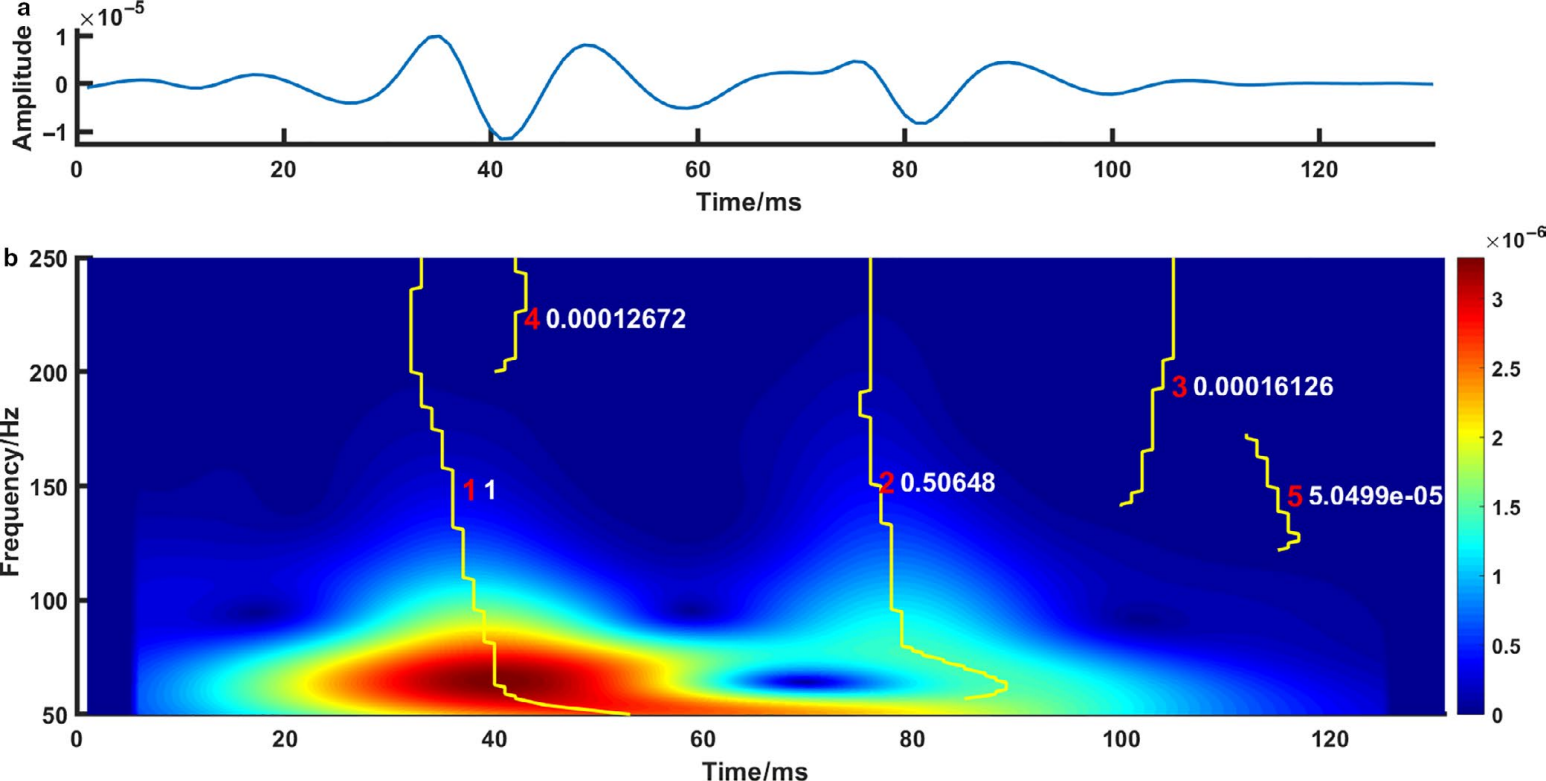
START algorithm for estimating A2‐P2 splitting interval. (a) A segmented S2 from animal experiment No.1 with A‐RV delay 150 ms and A‐LV delay 50 ms. (b) S‐transform amplitude spectrum and five ridges (solid yellow lines). The red number indicates the rank of each ridge. The white number indicates the weight factor of each ridge. The raw heart sound signal in panel A was processed with S‐transform to obtain a time–frequency–amplitude map in panel B, then the ridges of this map were identified and ranked according to their energies. The two ridges with the highest energies were used for calculating heart sound splitting interval. In this example, the START‐estimated S2 splitting interval is 44 ms

The START‐based detection of S2 splitting interval was performed on the S‐transform amplitude spectrum. It consists of two steps: ridge identification on the amplitude spectrum, and calculation of splitting interval from these ridges (Figure 2). 1) Ridge identification: A 50‐Hz highpass filter was applied to an S2, after which it underwent an S‐transform using mother wavelets with frequencies of 50 to 250 Hz, resulting in an amplitude spectrum. Local adjacent maxima of the amplitude spectrum were connected as a ridge. A ridge was used for further analysis if its frequency range covered more than 50 Hz. Doing so, in the example of Figure 2, 5 ridges were detected. 2) Subsequently, the importance of each ridge was graded using a weight factor which is the sum of products of amplitude and frequency of each ridge. This weight factor was indicative of the energy contained in each ridge. After finding all ridges, the weight factors were normalized to the highest one. Then two strongest ridges were considered to stem from A2 and P2. The A2‐P2 splitting interval was calculated as the median time between common frequencies of the two ridges.

### 2.3 Validation of START algorithm in S2 simulation model

The START algorithm was validated using artificial heart sounds, generated by a widely used nonlinear transient chirp signal model of S2 (Xu et al., (2000)). It first simulates A2 and P2, and then sums them up to obtain the entire S2. The A2 and P2 are simulated by the following equations, respectively:

(1) $$A2\left(t\right)=A_{A}\left(t\right)\sin\left(\varphi_{A}\left(t\right)\right)$$

(2) $$P2\left(t\right)=A_{P}\left(t\right)\sin\left(\varphi_{P}\left(t\right)\right)$$

The duration of each component, t, is defined as 0 ≤ t ≤ 60 ms. A and φ represent the amplitude and phase function of A2 and P2, respectively:

(3) $$A_{A}\left(t\right)=\mathrm{ampA}*\left(1‐e^{\frac{‐t}{8}}\right)*e^{\frac{‐t}{16}}*\sin\left(\frac{\pi t}{60}\right)$$

(4) $$A_{p}\left(t\right)=\mathrm{ampP}*\left(1‐e^{\frac{‐t}{8}}\right)*e^{\frac{‐t}{16}}*\sin\left(\frac{\pi t}{60}\right)$$

(5) $$\varphi_{A}\left(t\right)=24.3*t+451.4*\sqrt{t+1}$$

(6) $$\varphi_{p}\left(t\right)=21.8*t+356.3*\sqrt{t+1}$$

ampA and ampP represent the normalized amplitude of A2 and P2, respectively. In this study, we used a constant normalized amplitude of P2, so ampP = 1. Phase functions (5) and (6) control the frequency range of A2 and P2, respectively. To validate the START algorithm, we varied the splitting interval from 10 ms to 70 ms; ampA from 0.2 to 5.0; and signal‐to‐noise ratios (SNRs)from 10 to 30 decibels (dB).

### 2.4 Animal experiments

Open‐chest sacrifice pig experiments were performed in accordance with the Dutch Law on Animal Experimentation and the European Directive for the Protection of Vertebrate Animals Used for Experimental and Other Scientific Purposes. The protocol was approved by the Central Committee for Animal experiments (CCD) in The Netherlands and the Animal Experimental Committee of Maastricht University.

Five male adult pigs (weight: 64 ± 1 kg) were premedicated with prophylactic antibiotics (ampicillin 1000 mg I.V.) and thiopental (5–15 mg/kg, I.V.) for induction of general anesthesia (Heckman et al., 2020). Subsequently, they were intubated and mechanically ventilated, followed by maintenance of general anesthesia using rocuronium (0.1 mg/kg/h I.V.), sufentanyl (4–8 μg/kg/h I.V.), and propofol (2.5–10 mg/kg/h, I.V.). A left thoracotomy through the fifth intercostal space was performed to completely expose the epicardial surface. LV and RV pressure signals were acquired using 7F catheter‐tip manometers, inserted into the carotid artery and jugular vein, respectively. Pacing electrodes were transvenously placed in the right atrium, RV apex, and epicardially on the basal posterolateral wall of LV. Complete atrioventricular (AV) block was induced by radiofrequency ablation.

Heart sounds were collected by a triaxial accelerometer with a sample rate of 1000 Hz, positioned on the anterior RV base. This position was chosen because it was close to the pulmonic and aortic valves. After creation of AV‐block, biventricular pacing was used with a fixed atrial (A) to RV (A‐RV) pacing delay (150 ms) and varying A to LV (A‐LV) pacing delays (50 ms to 250 ms), using 25 ms in the 1st experiment or 50 ms per step in the remaining 4 experiments.

During each pacing setting, ECG and hemodynamic signals were collected for 20–30 seconds using the IDEEQ data acquisition system (IDEE Maastricht University / Maastricht Instruments BV). Accelerometer signals were collected using a custom‐made data acquisition system. Hemodynamic signals and accelerometer signals were aligned using a synchronous pulse signal. Hemodynamic analysis was performed using the IDEEQ software, developed at Maastricht University.

### 2.5 Calculation of interventricular relaxation dyssynchrony (IRD)

**Figure 3:**
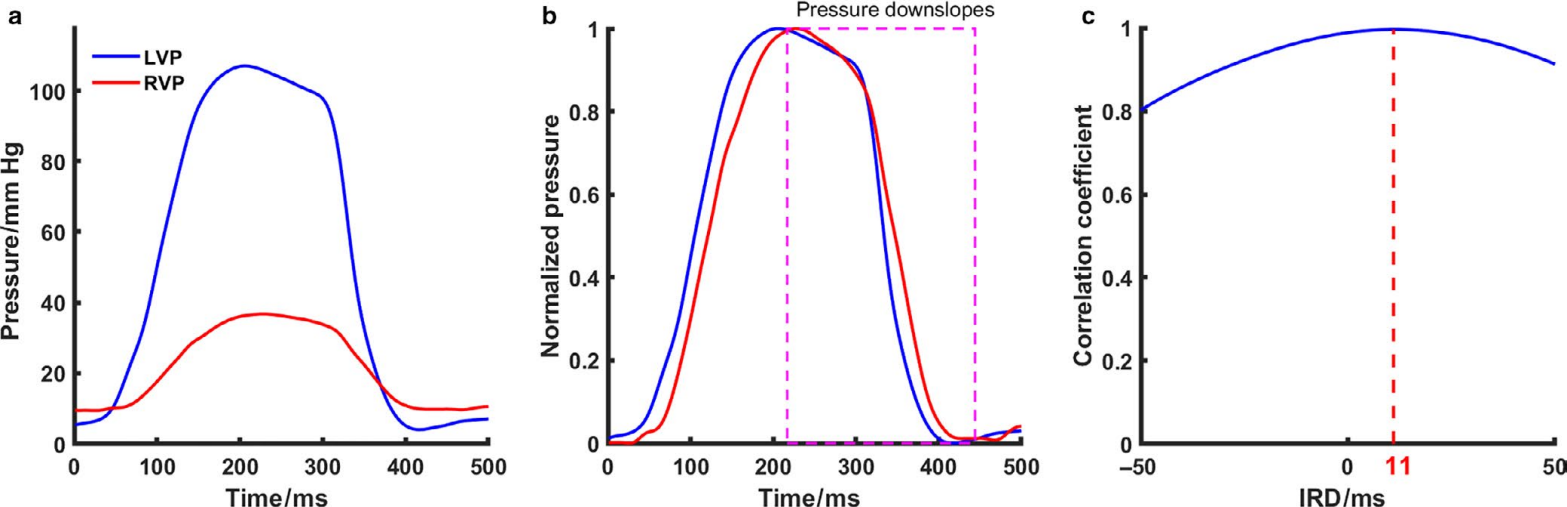
Demonstration of interventricular relaxation dyssynchrony calculation. (a) Raw LV and RV pressures from animal experiment No.1 at A‐RV delay 150 ms and A‐LV delay 50 ms. (b) Normalized LV and RV pressure, with their downslopes used for correlation calculation. (c) Correlation coefficient curve indicating an interventricular relaxation dyssynchrony of 11 ms. Raw LV and RV pressures in the panel A were normalized in the panel B. The downslopes of the normalized pressures were cross‐correlated to obtain the correlation coefficients in the panel C. The time when the correlation coefficient was the highest was used as an estimate of interventricular relaxation dyssynchrony. LVP =left ventricular pressure; RVP =right ventricular pressure

IRD was defined as the time difference between the downslopes of LV and RV pressure curves (Figure 3). Pressure data were filtered using a second‐order Butterworth bandpass filter with the range of 0.5–40 Hz. After normalizing both pressure curves to the range of 0 to 1, IRD was determined as the time shift between LV and RV pressure, required to achieve the highest correlation coefficient. Positive IRD indicates the LV downslope being earlier than the RV downslope. This approach is similar to that used in our previous study to determine interventricular mechanical dyssynchrony during isovolumic contraction phase (Verbeek et al., 2002).

### 2.6 Heart sound signal processing

From data acquired during the animal experiments, a combined accelerometer signal was calculated from the raw signals of X, Y, and Z directions, and double integrated to obtain a displacement signal. A second‐order Butterworth bandpass filter of 50–250 Hz was applied. Locations of S1 and S2 were identified with reference to lead II electrocardiogram (ECG). To reduce the effect of any sudden vibrations and background noises on manual identification, we overlapped all heartbeats and calculated a median heart sound signal as a reference. Premature ventricular contraction beats and their two subsequent heartbeats as well as heartbeats with too much noise were discarded. Finally, the resulting S2 signal was processed with the START algorithm to obtain splitting interval.

### 2.7 Comparison with existing heart sound splitting detection algorithms

To clarify the role of our START algorithm in comparison with other existing heart sound splitting detection algorithms, we searched the PubMed database using terms of “heart sound” and “split*” on October 27, 2020. Date of publication was from the year 1970. Each publication was first judged by title and abstract. Publications including reviews, letters, and case reports were excluded. Potential articles were further screened for full text. The search approach was complemented by consulting experts in the field for potential related studies. We included original studies which described heart sound splitting detection algorithms. The following information was extracted from each eligible article: first author name, year of publication, brief description of the algorithm, whether the algorithm works on a single heartbeat, and whether a validation study was performed. Validation study could be any of the following: 1) validation of the algorithm in various splitting intervals; 2) validation of the algorithm in various A2/P2 amplitude ratios; or 3) validation of the algorithm in various SNRs.

### 2.8 Statistical analysis

Hemodynamic data and summary of correlation data were expressed as mean and standard deviation. S2 splitting interval and IRD were expressed as median (25th percentile, 75th percentile) to reduce the potential effect of respiration on our analysis. Pearson's correlation was calculated between START‐estimated splitting interval and simulated splitting interval as well as between estimated splitting interval and IRD during animal experiments. Spearman's rank correlation was calculated between splitting interval and A‐LV delay as well as between IRD and A‐LV delay. A P value less than 0.05 was assumed to indicate a statistically significant difference. All statistical analyses were performed using MATLAB R2018b and Stata/MP 14.0.

## 3 RESULTS

### 3.1 Validation of START algorithm in simulated signals

**Figure 4:**
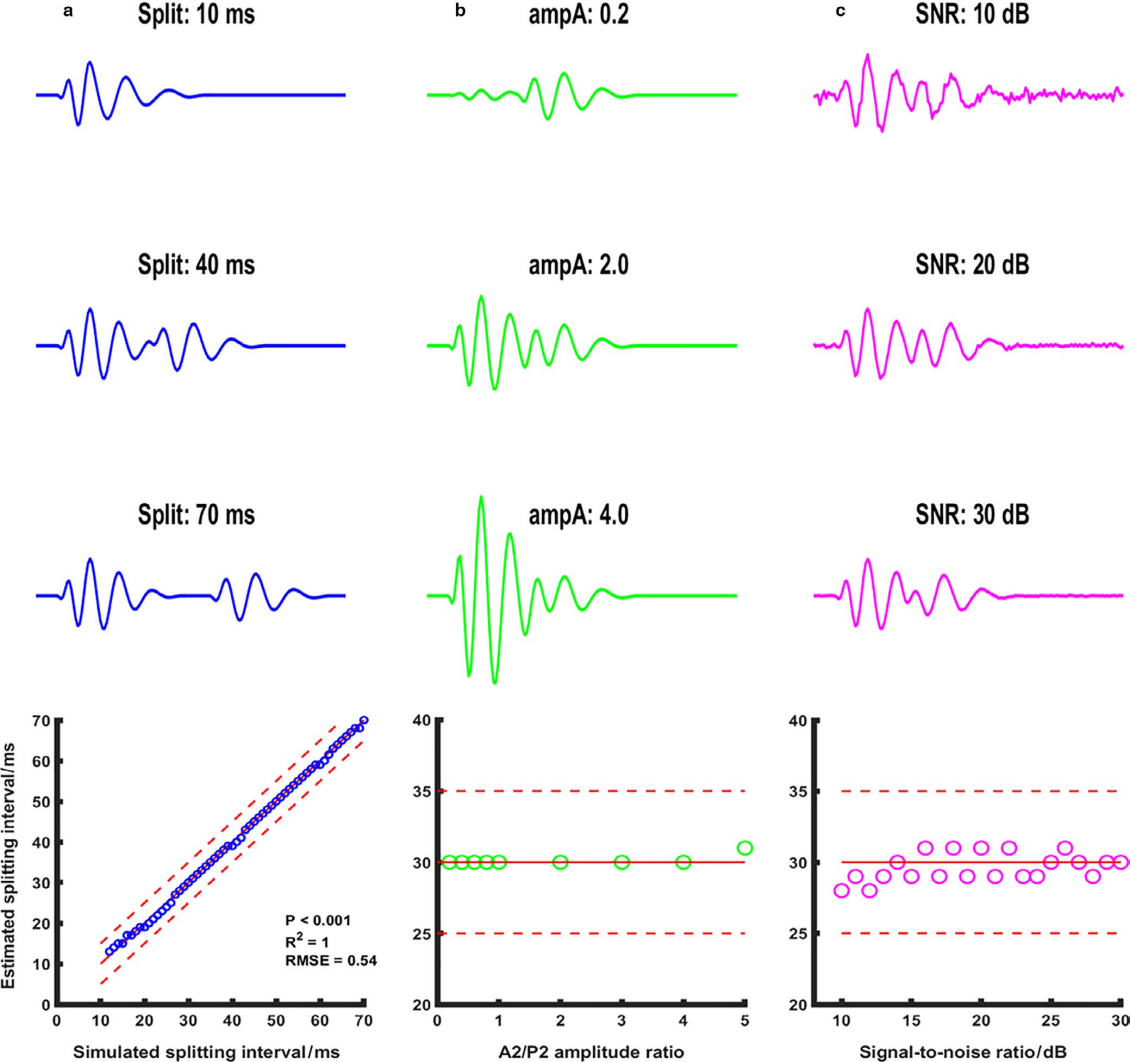
START algorithm in simulated second heart sounds. Red solid line indicates line of identity in panel A and 30 ms in panels B and C. Red dash lines are 5‐ms upper and lower boundaries of the expected values. Panel A (in blue) demonstrated three representative simulated heart sounds of splitting intervals 10 ms, 40 ms, and 70 ms, whereas the bottom figure showed the relationship between simulated and START‐estimated splitting intervals from 10 ms to 70 ms. Pearson's correlation was calculated. Panel B (in green) demonstrated three representative simulated heart sounds with normalized A2 amplitudes of 0.2, 2.0, and 4.0 when the normalized P2 amplitude was fixed at 1.0. In all these cases, the simulated splitting intervals were fixed at 30 ms, and the bottom figure showed the errors of splitting interval estimation using our proposed START algorithm. Panel C (in magenta) demonstrated three representative simulated heart sounds of signal‐to‐noise ratios of 10, 20, and 30 dB. In all these cases, the simulated splitting intervals were fixed at 30 ms, and the bottom figure showed the errors of START‐based splitting interval estimation for signal‐to‐noise ratios from 10 dB to 30 dB at step of 1 dB. ampA =normalized amplitude of aortic component of S2; dB =decibel; RMSE =root mean square error; SNR =signal‐to‐noise ratio

When using the simulated heart sound signals, the START algorithm had a high accuracy at a wide range of splitting intervals (10–70 ms; R^2^ = 1, *p* < .001) (Figure 4a). Mean estimation error was 0.5 ms in this range. When fixing the normalized P2 amplitude at 1 and varying the A2 amplitude from 0.2 to 5, the splitting estimation was stable for the ampA/ampP ratios below 4 and slightly increased by 1 ms at ampA/ampP ratio of 5 (Figure 4b). From SNRs of 10 dB to 30 dB, the estimated splitting values fluctuated around the expected value by 1 to 2 ms (Figure 4c).

### 3.2 Animal experiments

Table 1 summarizes the hemodynamic data of the five pigs with AV block, measured during simultaneous RV and LV pacing with an AV delay of 150 ms.

**Table 1:** Summary of hemodynamics (n = 5)^a^

Variables	Values
Heart rate (bpm)	94 ± 23
Systolic blood pressure (mmHg)	99 ± 21
Diastolic blood pressure (mmHg)	71 ± 19
LV dp/dt max (mmHg / sec)	1337 ± 206
LV dp/dt min (mmHg / sec)	−1685 ± 447
RV dp/dt max (mmHg / sec)	337 ± 25
RV dp/dt min (mmHg / sec)	−358 ± 75

bpm = beats per minute; LV = left ventricle; RV = right ventricle.

^a^ Data were measured during biventricular pacing with AV delay of 150 ms.

**Figure 5:**
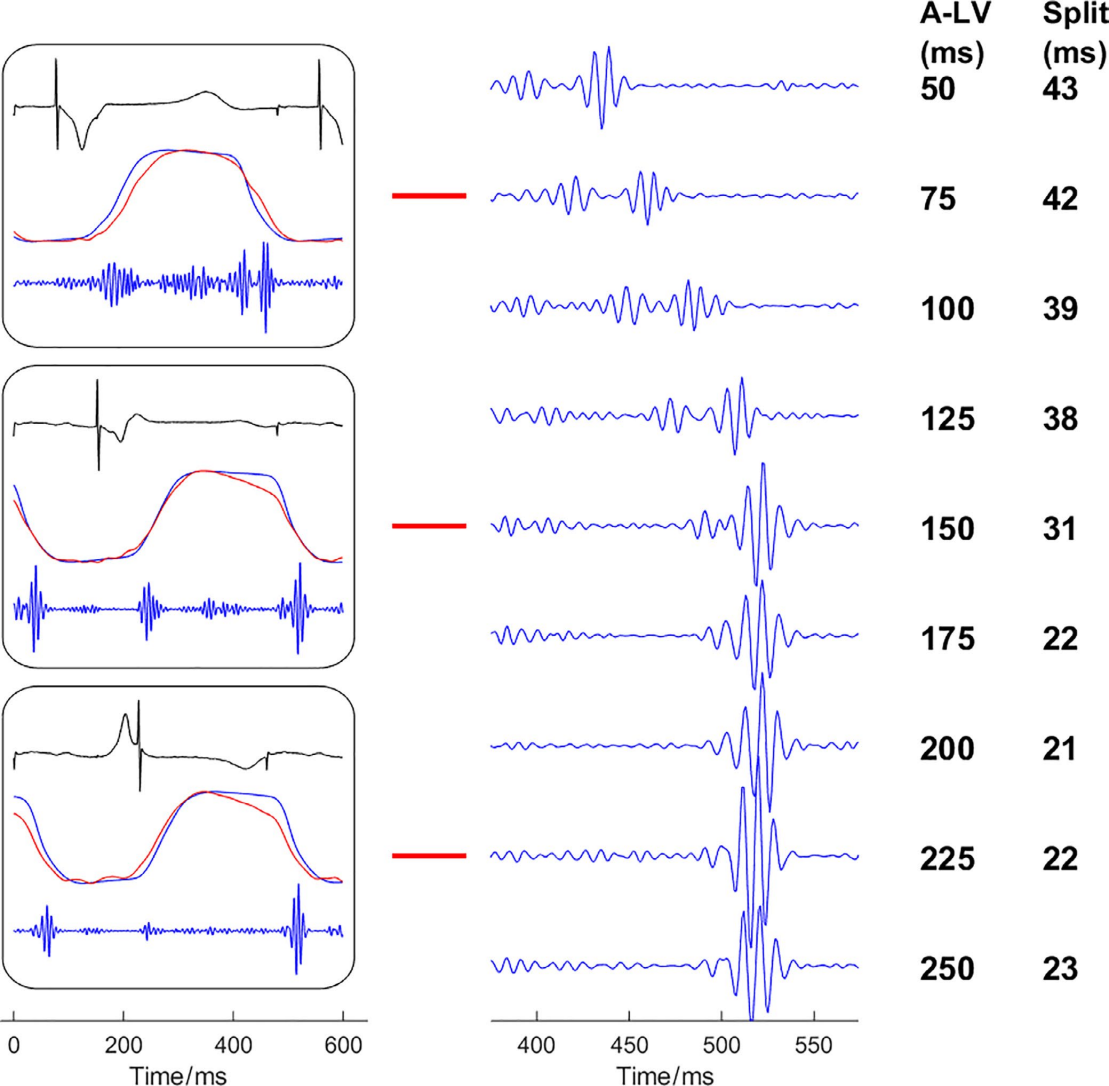
Representative examples of second heart sound splitting at various paced interventricular delays. When A‐RV delay was fixed at 150 ms, A‐LV delays were varied from 50 ms to 250 ms with 25 ms per step. Representative heart sounds from experiment No.1. In the left panels, three situations with A‐LV delays of 75, 150, and 225 ms are shown with recordings of electrocardiogram, LV pressure (blue), RV pressure (red), and heart sounds. In the right panel, S2 is shown at higher temporal resolution. Note that S2 splitting decreases from A‐LV delays of 50 ms to 150 ms, but remains virtually constant at longer A‐LV delays. S1 = the first heart sound; S2 = the second heart sound; LV =left ventricle; RV =right ventricle; A‐LV =right atrium to left ventricle paced delay.

**Figure 6:**
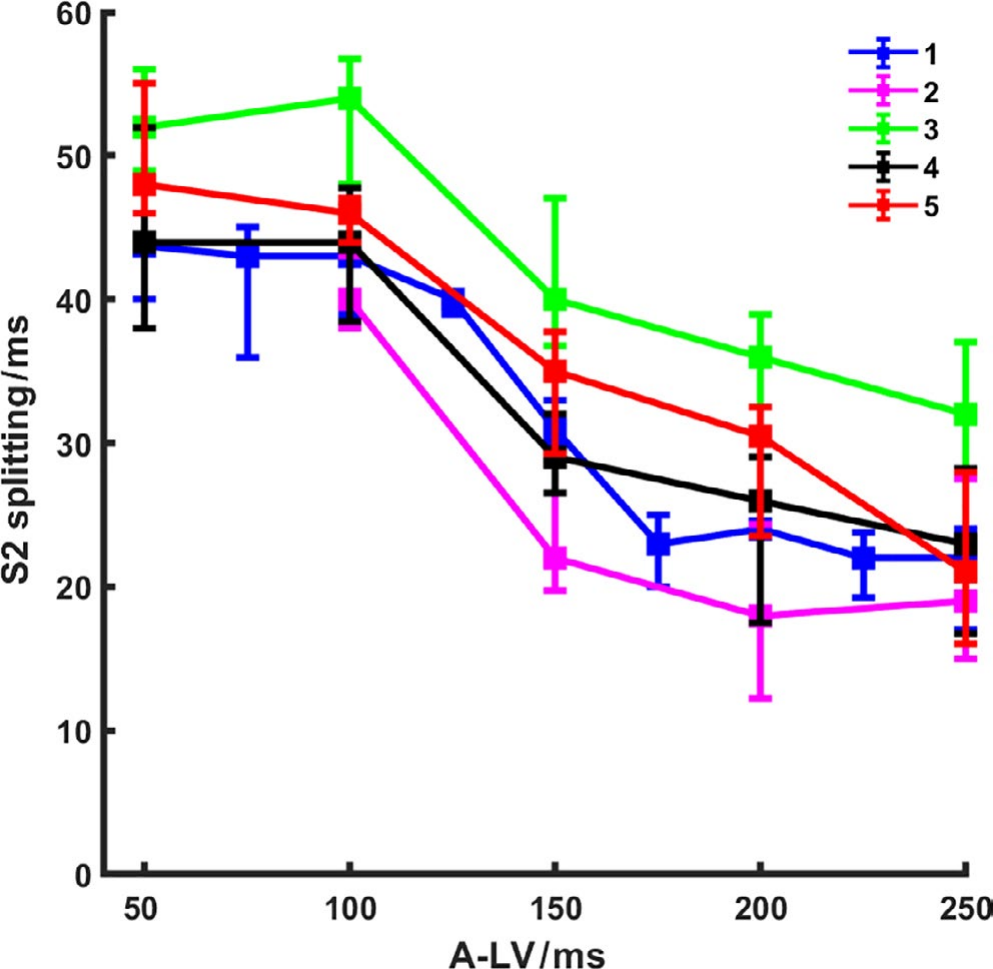
Second heart sound splitting interval as a function of atrio‐left ventricular stimulation interval (A‐LV). Each color represents a different animal. Median values and 25–75% percentiles of all beats at each A‐LV interval are presented (A‐RV delay was fixed at 150 ms). From experiments No. 1 to 5, the median number of heartbeats was 38, 43, 34, 29, and 33, respectively, for each A‐LV delay group. Spearman's rank correlation R^2^ value was 0.71, 0.41, 0.63, 0.64, and 0.72 for experiments 1 to 5, respectively (*p* < .001 in all experiments). S2 = second heart sound

The left panels of Figure 5 show examples of the measurements of RV and LV pressure as well as heart sounds at three A‐LV delays (75 ms, 150 ms, and 225 ms) when A‐RV delay was fixed at 150 ms. Note that at an A‐LV of 75 ms LV pressure rises before RV pressure and that this is accompanied by a clear S2 splitting. S2 splitting becomes smaller with longer A‐LV delays, that is more simultaneous LV and RV activation (A‐LV 150 ms) and earlier RV activation (A‐LV 225 ms). Note that the amplitudes of S2 also became higher than A2 and P2 merged. The most significant changes in A2 and P2 occurred from A‐LV delay of 100 ms to 200 ms. This is further demonstrated in all five experiments in Figure 6. S2 splitting interval significantly decreased from low to high A‐LV intervals (representing changes from LV pre‐excitation to RV pre‐excitation) (all *p* < .001).

**Figure 7:**
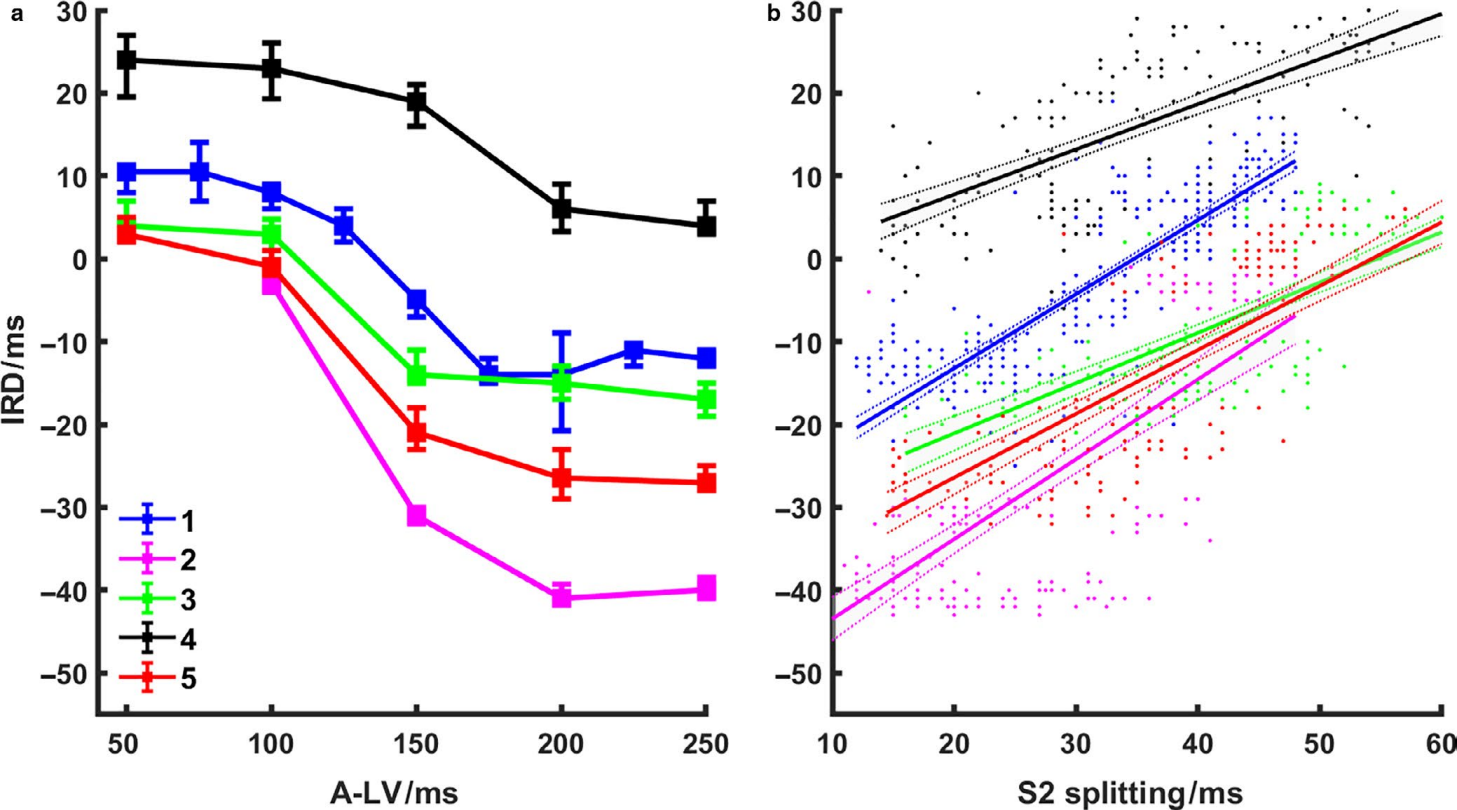
(a) Changes in interventricular relaxation dyssynchrony (IRD) as a function of A‐LV delay (n = 5). A‐RV delay was fixed at 150 ms. From A‐LV delay of 50 ms to 250 ms at step of 50 ms, the overall IRD was 8 (4,12) ms, 2 (−2,8) ms, −14 (−24, −5) ms, −20 (−39, −11) ms, and −18 (−28,‐11) ms, respectively. Data are presented as median (25 and 75 percentile). Spearman's rank correlation R2 value was 0.72, 0.73, 0.75, 0.64, and 0.82 for experiments 1 to 5, respectively (*p* < .001 for each experiment). (b) Relation between IRD and S2 splitting. Each point represents a heartbeat. Each color represents an experiment, the same as in Figure 6. Equations and Pearson's correlation coefficients of the correlation plots are summarized in Table 2). A‐LV =right atrium to left ventricle paced delay; S2 = the second heart sound

Figure 7a showed decreasing IRDs as A‐LV delays changed from 50 ms to 250 ms, when A‐RV delay was fixed at 150 ms (all *p* < .001). It is noteworthy that like S2 splitting interval in Figure 6, the most significant changes of IRD occurred from A‐LV delay of 100 ms to 200 ms. Figure 7b demonstrated that for each animal experiment, there was a strong correlation between START‐estimated S2 splitting intervals and invasively measured IRDs (all *p* < .001). Table 2 presented a summary of the results of linear curve fitting and correlation coefficient between S2 splitting and IRD.

**Table 2:** Pearson's Correlation of second heart sound splitting interval with interventricular relaxation dyssynchrony

Experiment No.	Number of heartbeats	Slope	Intercept	R^2^	*p* value
1	346	0.90	−31	0.72	<.001
2	172	0.96	−53	0.54	<.001
3	163	0.61	−33	0.56	<.001
4	143	0.55	−3	0.49	<.001
5	150	0.77	−42	0.63	<.001

### 3.3 Comparison of START algorithm with other splitting detection algorithms

Searching the PubMed database using “heart sound” and “split*” resulted in 120 publications. After excluding six reviews, three letters and 35 case reports, the remaining 76 publications were checked for description of heart sound splitting detection algorithm. Two publications were recommended by experts in the field (Chen et al., 2018; Tang et al., 2017). Finally, 13 publications on 12 splitting detection algorithms were included for analysis (Table 3).

**Table 3:** Summary of publications of heart sound splitting detection algorithm

First author [Ref.]	Year	Description	Detection threshold (ms)	Work on a single heartbeat? (Y/N)	Validation? (Y/N)
Xu et al. (2002)	2002	Nonlinear transient chirp signal modeling	‐‐	Y	N
Popov et al. (2004)	2004	Gaussian chirplet modeling	‐‐	Y	N
Nigam & Priemer (2006)	2006	Blind source separation	≥0	N	Y
Debbal & Bereksi‐Reguig (2006, 2007)	2006	Continuous wavelet transform	‐‐	Y	N
Yildirim & Ansari (2007)	2007	Smoothed Wigner–Ville distribution	≥20	Y	Y
Al‐Naami et al. (2010)	2010	Continuous wavelet transform and support vector machine	‐‐	Y	N
Hamza & Debbal (2013)	2013	Hilbert transform envelope	‐‐	Y	N
Djebbari & Bereksi‐Reguig, 2013)	2013	Reassigned smoothed pseudo Wigner–Ville distribution	≥30	Y	Y
Thiyagaraja et al. (2014)	2014	Continuous wavelet transform	‐‐	Y	N
Barma et al. (2015)	2015	Hilbert vibration decomposition and reassigned smoothed pseudo Wigner–Ville distribution	≥20	Y	N
Tang et al. (2017)	2017	Respiration‐modulated splitting measurement	≥25	N	Y
Chen et al. (2018)	2018	Blind source separation	‐‐	N	N
Proposed method	2020	S‐transform	≥10	Y	Y

Early efforts were focused on using modeling approaches to simulate heart sound morphologies and further to extract splitting interval (Popov et al., 2004; Xu et al., 2002). Blind source separation was proposed only for multiple‐channel simultaneous recordings (Chen et al., 2018; Nigam & Priemer, 2006). Many later studies employed CWT and SWVD to identify A2 and P2 on time–frequency spectrum (Al‐Naami et al., 2010; Barma et al., 2015; Debbal & Bereksi‐Reguig, 2006, 2007; Djebbari & Bereksi‐Reguig, 2013; Thiyagaraja et al., 2014; Yildirim & Ansari, 2007). However, both methods relied on visual identification of two well‐separated components of heart sound on time–frequency spectrum, making the detection threshold exceeding 20 ms. Moreover, the SWVD method was complicated by cross‐terms which interfered with the identification of A2 and P2. Validation of splitting detection algorithm in various splitting intervals was provided only in 4 studies (Djebbari & Bereksi‐Reguig, 2013; Nigam & Priemer, 2006; Tang et al., 2017; Yildirim & Ansari, 2007). Validation of algorithm in various SNRs was provided only in one study (Yildirim & Ansari, 2007). No studies provided validation on various A2/P2 amplitude ratios.

## 4 DISCUSSION

We introduced a time–frequency‐based START method to estimate S2 splitting, validated it with simulated heart sounds, and employed it to observe S2 splitting in porcine experiments. Major findings of our study are as follows: 1) the START algorithm for estimating S2 splitting interval is accurate in a wide range of splitting intervals, A2/P2 amplitude ratios, and SNRs; and 2) START‐estimated S2 splitting interval is significantly correlated with paced interventricular delays and IRD, though no correlations are observed for long A‐LV delays in porcine models probably because of the fact that the LV is activated even before the moment of pacing. To our knowledge, our study is the first to investigate the possibility of using a heart sound indicator to monitor interventricular dyssynchrony in CRT‐like settings. As a simple measurement, heart sound is likely to serve as a promising real‐time monitoring approach for patients with interventricular dyssynchrony.

### 4.1 Comparison of START algorithm with other splitting detection methods

Distinguishing the time difference between the two components of S2, that is, A2 and P2, has long been a challenge because of their overlap within a short period of time. Our proposed algorithm works for a small splitting interval down to 10 ms, for various A2/P2 amplitude ratios and for low SNRs. Previous studies using modeling approaches to extract splitting interval assumed a standard template of heart sound which remains debatable (Popov et al., 2004; Xu et al., 2002). Blind source separation method required at least four simultaneous recordings to obtain a satisfactory splitting estimation (Nigam & Priemer, 2006). The respiration‐modulated splitting measurement method required a continuous recording of at least 200 heartbeats to obtain a relatively robust estimation (Tang et al., 2017). Our proposed START algorithm does not rely on theories about genesis, transmission, or statistical characteristics of heart sound, making it adaptable to heart sounds of various shapes from different individuals. This is supported by the consistency of decreasing trends of S2 splitting during varying paced interventricular delays in the five pigs. The START algorithm works on a single‐sensor single heartbeat, enabling it to be applicable to a short heart sound recording. Although previous time–frequency algorithms including CWT and SWVD could also work on a single heartbeat, they relied on two well‐separated components of time–frequency spectrum to label A2 and P2 (Al‐Naami et al., 2010; Barma et al., 2015; Debbal & Bereksi‐Reguig, 2006, 2007; Djebbari & Bereksi‐Reguig, 2013; Thiyagaraja et al., 2014; Yildirim & Ansari, 2007). Moreover, for SWVD method, the clear identification of A2 and P2 on time–frequency spectrum was interfered by the unavoidable introduction of cross‐terms during signal processing. Our proposed START algorithm improves the efficiency and accuracy of existing time–frequency algorithms by automatically tracking the ridges of A2 and P2 on time–frequency spectrum. This avoids the bias of identifying A2 and P2 by human eyeballs, especially for low splitting intervals.

Furthermore, lack of validation is likely to render many existing splitting detection algorithms to be unclear for challenging conditions such as extremely low or high A2/P2 amplitude ratios, or low SNRs. Among the various algorithms proposed to assess S2 splitting, our algorithm is unique also in the sense that it has been evaluated not only in simulated heart sounds of various conditions but more importantly, in heart sounds acquired from an *in vivo* porcine model of varying VV delays. In animal studies, the proposed method captured all the decreasing trends of S2 splitting within a narrow range from around 40 ms to around 20 ms during changing paced interventricular intervals.

### 4.2 Relationship between S2 splitting and IRD

Our study demonstrates a good correlation between S2 splitting interval and IRD, confirming its value as a noninvasive indicator of interventricular dyssynchrony. Using a highly accurate splitting detection algorithm and an animal model with adjustable paced interventricular delays, our study for the first time provides direct experimental evidence to an old assumption that S2 splitting is associated with synchronism at the end of the ejection period of the two ventricles (Wolferth & Margolies, 1935). Current methods of evaluating IRD mainly rely on echocardiography which is time‐consuming, operator‐dependent, and is commonly performed in the recumbent position (Dreger et al., 2012). Moreover, the echocardiography‐derived IRD is confined to a few cardiac cycles and thus cannot be used for continuous monitoring of the patient's status. In contrast, S2 splitting interval derived from heart sounds may be collected continuously using sensors on the chest or incorporated in implantable devices.

One initially surprising observation of this study is that while there was a clear S2 splitting during LV pre‐excitation, no reverse splitting was observed during RV pre‐excitation. At long A‐LV delays, the LV was already activated by means of myocardial conduction coming from the paced RV. The explanation for this observation may also be found in the fact that in general, ejection time is shorter in the LV than in the RV. In the synchronously activated heart, this leads to earlier aortic than pulmonic valve closure and consequently an earlier onset of A2 than P2. LV pre‐excitation most likely increases the time interval between aortic and pulmonary valve closure and thus S2 splitting. RV pre‐excitation in its turn shifts the pulmonic valve closure to earlier time points, but apparently not before aortic valve closure in our porcine animal model. This finding implies that S2 splitting interval is more sensitive to detect LV than RV pre‐excitation. This is further supported by findings of Xiao *et al*. who showed that 88% (21/24) of patients with RV pacing did not show any reversed splitting of S2 (Xiao et al., 1993). In CRT the aim is to synchronize contraction of the two ventricles. As part of this approach, pacemaker settings can be adjusted to vary the activation time of RV and LV. The data of this study show that S2 splitting can be used to detect LV pre‐excitation associated with pacing. Although respiration may also affect the detection, in the current experimental setting, with open chest and mechanical ventilation, respiratory variations were negligible.

### 4.3 Comparison with other heart sound measures

Several other heart sound‐based indicators have been used for CRT optimization. These include electromechanical activation time (time between onset of electrical activation and onset of S1), left ventricular systolic time (time difference between S1 and S2), and S3 strength (Taha et al., 2010; Toggweiler et al., 2006, 2007; Zuber et al., 2008). The only heart sound approach that has progressed to clinical application is the SonR algorithm, which uses the amplitude of S1 as an indicator. This algorithm is used for repetitive and automated optimization of CRT. The SonRtip Lead and Automatic AV‐VV Optimization Algorithm in the Paradym RF SonR CRT‐D (RESPOND CRT) Trial showed that SonR‐guided optimization of atrioventricular and interventricular timings significantly improves CRT responder rate and reduces risk of heart failure hospitalization as compared with the conventional, inconsistently applied optimization (Brugada et al., 2014, 2016).

While these parameters mainly reflect contractility, the START‐based S2 splitting is the only indicator of interventricular dyssynchrony from heart sound so far and may therefore be of additional value on top of the aforementioned indicators. It can be imagined that S2 splitting can be used to guide pacing lead location during CRT implantation as well as in CRT optimization during follow‐up, either manually during outpatient visits or, when accelerometers are incorporated in pacing leads and or pacemakers, in ambulatory fashion.

## 5 LIMITATIONS

Translating results from animal experiments to clinical applications should be done with caution. First of all, the conduction system in the pigs differs to some extent from that of humans, resulting in narrower QRS complexes during ventricular pacing. This likely also results in smaller interventricular dyssynchrony. The fact that START algorithm can detect the small S2 splitting intervals in pigs suggests therefore, that this certainly will be possible in humans. Second, the pigs had normal cardiac function, whereas humans eligible for pacing treatments may have depressed cardiac function. Third, the number of experiments was small and measurements were performed during anesthesia and with an open chest.

## 6 CONCLUSIONS

The proposed START algorithm is accurate in estimating S2 splitting under a wide range of conditions as shown by heart sound simulation. Our pilot animal experiment in the AV block porcine model demonstrates that the START‐based estimated S2 splitting interval can be used as an indicator of interventricular dyssynchrony. The estimated S2 splitting is well correlated with paced interventricular delays and with invasively measured IRD.

## CONFLICT OF INTEREST

We declare no competing interests. R.C. is an employee of Medtronic.

## AUTHORS’ CONTRIBUTIONS

H.L., P.W., L.H., M.K., R.C., and F.P. conceived and designed research; H.L., P.W., L.B., and M.K. performed experiments; H.L. analyzed data, prepared figures, and drafted manuscript; H.L., P.W., M.S., R.C., T.D., and F.P. interpreted results of experiments; H.L., R.C., T.D., L.H., and F.P. edited and revised manuscript; H.L., P.W., M.S., L.H., M.K., R.C., T.D., and F.P. approved final version of the manuscript.
